# Interactions between egocentric and allocentric spatial coding of sounds revealed by a multisensory learning paradigm

**DOI:** 10.1038/s41598-019-44267-3

**Published:** 2019-05-27

**Authors:** Giuseppe Rabini, Elena Altobelli, Francesco Pavani

**Affiliations:** 1 Center for Mind/Brain Sciences (CIMeC), University of Trento, Trento, Italy; 2 Department of Psychology and Cognitive Sciences (DiPSCo), University of Trento, Trento, Italy; 3 IMPACT, Centre de Recherche en Neuroscience Lyon (CRNL), Bron, France

**Keywords:** Human behaviour, Perception

## Abstract

Although sound position is initially head-centred (egocentric coordinates), our brain can also represent sounds relative to one another (allocentric coordinates). Whether reference frames for spatial hearing are independent or interact remained largely unexplored. Here we developed a new allocentric spatial-hearing training and tested whether it can improve egocentric sound-localisation performance in normal-hearing adults listening with one ear plugged. Two groups of participants (N = 15 each) performed an egocentric sound-localisation task (point to a syllable), in monaural listening, before and after 4-days of multisensory training on triplets of white-noise bursts paired with occasional visual feedback. Critically, one group performed an allocentric task (auditory bisection task), whereas the other processed the same stimuli to perform an egocentric task (pointing to a designated sound of the triplet). Unlike most previous works, we tested also a no training group (N = 15). Egocentric sound-localisation abilities in the horizontal plane improved for all groups in the space ipsilateral to the ear-plug. This unexpected finding highlights the importance of including a no training group when studying sound localisation re-learning. Yet, performance changes were qualitatively different in trained compared to untrained participants, providing initial evidence that allocentric and multisensory procedures may prove useful when aiming to promote sound localisation re-learning.

## Introduction

Accurate processing of sound source coordinates is central to our ability to perceive the 3D structure of the surrounding auditory scene^1–3^, discern signal from noise^4^ and orient attention in space^5,6^. Sound localisation relies on the interpretation of auditory cues (interaural time and level differences, as well as monaural spectral cues) deriving from the interactions between sound waves, the head and external ears^7,8^. Initial coding of sound position occurs in head-centred coordinates^9^, in an *egocentric* reference frame dependent on the head position on the trunk and listener’s position in the environment. However, efficient interactions with the auditory environment, require encoding of sound position also in a reference frame that remains stable when our head and our body change position. Such reference frame is termed *allocentric* (or world-centred) and it encodes the position of sounds with respect to other sound sources or external landmarks in the multisensory environment. This allocentric reference frame allows judgements on the relative positions of sounds, independent of the listener’s position.

Coding of auditory space in different reference frames has previously been investigated in humans using electrophysiology, taking advantage of the Mismatch Negativity (MMN) response. In two studies using virtual sounds delivered through headphones^10^ or free-field sounds^11^, Altmann and colleagues found evidence for head-centred (craniocentric) coding at the early processing stage corresponding to the MMN. However, a similar study conducted with free-field sounds by Schechtman and colleagues found that head-dependent and head-independent^11^ sound representations may in fact coexist, already at the processing stage captured by the MMN. The latter result is in agreement with research on ferrets, indicating that a majority of neurons in the auditory cortex is tuned to egocentric sound representation, and yet a restricted subsample of cells showing head-independent position coding also exists^10^.

To the best of our knowledge, only one study has examined egocentric and allocentric sound coding using behavioural methods, focusing however on a special population. Studying sighted and blind individuals, Gori and colleagues^12^ showed that congenitally blind people are highly accurate when asked to localise single sounds (an egocentric task), but they are impaired in a task that require a judgment on the relative distance between pairs of sounds (i.e., the auditory space bisection task, an allocentric task). This study revealed that sound localisation abilities in allocentric and egocentric reference frames can dissociate, at least in visually impaired people. In general, however, the issue of dissociation vs. interaction of egocentric and allocentric sound coding in humans remained largely unexplored. In the present study, we aimed to investigate this interaction in behaviour by testing if a training of sound localisation abilities performed in allocentric coordinates (auditory space bisection task) can improve spatial hearing performance in a pointing to sound task performed in egocentric coordinates.

Research on sound localisation training over the last decades has consistently revealed the auditory system plasticity when dealing with altered auditory cues^13^. Studies in humans^14–18^ as well as other animals^19^, showed that learning new correspondences between auditory cues and coordinates in space is possible, particularly when training paradigms are employed. The theoretical relevance of these findings resides in the identification of a perceptual ability which remains plastic beyond sensitive periods in the early stages of development^20^. Moreover, these observations have great clinical implications, as they open the possibility of training spatial hearing in adults with hearing loss, when binaural hearing is restored through hearing aids or cochlear implants. Several learning paradigms aimed at improving spatial hearing have adopted a multisensory approach^16^. This approach builds on the notion that multisensory training can promote unisensory learning^21^. In the case of audio-visual training, the hypothesis is that the brain can calibrate the association between auditory cues and spatial locations in external space, taking advantage of the more reliable visuo-spatial information. Support for this approach emerged from converging results in monaurally plugged hearing humans^22^ and in ferrets with bilateral cochlear implants^23^. To date, however, all audio-visual training paradigms have been implemented in egocentric reference frame^16^, with no attempt to exploit the same principles in the context of allocentric coding of sounds.

In audio-visual training paradigms the two multisensory events originate from identical positions in space (i.e., auditory and visual stimuli are spatially congruent). This perceptual coupling has been accomplished using either simultaneous or delayed multisensory stimulation. For instance, the audio-visual training developed by Strelnikov and collegues^22^ consisted in single sounds delivered in free-field, and paired with simultaneous and spatially congruent visual events presented using LEDs. Participants were asked to localise each sound, using a laser pointer controlled by a central knob. Notably, in order to avoid a complete reliance on visual information, the training session comprised occasional (15%) auditory-only stimulations. Differently, Majdak and co-workers^24^, implemented a virtual reality setup in which participants localised a single sound delivered though headphones in virtual auditory space, using a virtual reality laser pointer. No concomitant visual stimulation was delivered at this first stage. After the response, a red rotating cube was presented as feedback, to indicate the actual sound position (delayed spatially congruent stimulation). Finally, the sound was replayed once more from the same location, this time spatially and temporally coupled with the visual stimulus. Note that in both training studies, target sounds were localised with respect to the participant’s body and required, in this sense, egocentric coding. An allocentric training would have require instead localisation of the sound with respect to a different object or landmark, or alternatively, the presentation of multiple sounds in different positions with a subsequent evaluation of their relative spatial location.

**Figure 1:**
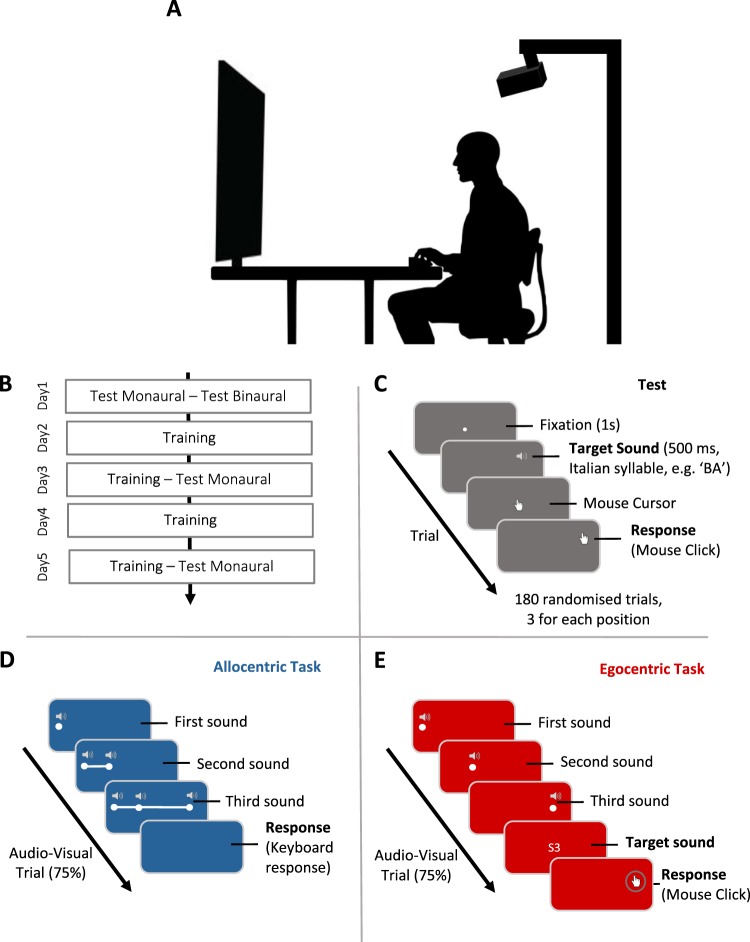
Apparatus, overall experimental procedure and tasks. (**A**) Participants sat in front of a matrix (5 × 12) of loudspeakers mounted on a rectangular panel, on which visual stimuli were projected when appropriate. Response were collected through mouse cursor or keyboard. (**B**) The experiment lasted five consecutive days, with testing sessions at day 1, day 3 and day 5, and training sessions from day 2 to day 5. (**C**) During testing sessions, participants completed an egocentric sound localisation task. In each trial, a spoken Italian syllable was presented from one speaker of the stimulation matrix and participants indicated the source of the sound by left-clicking with the mouse cursor in the correspondent position. (**D**) The AlloT Group performed the acoustic space bisection task. Three consecutive sounds (white noise) were presented at different horizontal positions and participants indicated if the second sound was closer to the first or third presented sound. During audio-visual trials (75% of total) a white dot appeared on screen to mark the exact position of each sound, together with lines connecting the dots to depict the spatial relationships between sounds. (**E**) The EgoT group received exactly the same audio-visual stimulation (with the only exception of lines connecting the dots) but was instructed to localise only one of the three presented sounds using the mouse.

In the present work, we directly compared the efficacy of two multisensory trainings – one based on *allocentric* spatial processing, the other based on *egocentric* spatial processing – in promoting re-learning of correspondences between auditory cues and space. Following previous works, we altered spatial hearing by plugging one ear. Monaural listening deteriorates sound localisation performance^25–27^ and proved an effective model for the study of auditory space re-learning^28^. One group of participants was trained using a multisensory (audio-visual) allocentric task, modelled on the auditory space bisection proposed by Gori and collaborators^29^. Three bursts of white noise were presented in sequence, paired with spatial and temporally matching visual stimuli. Participants in this group judged the relative position of the central sound with respect to the other two (see Fig. 1D). A second group of participants was trained using exactly the same audio-visual triplets, but was asked to perform an egocentric localisation task. A written instruction presented at the end of the trial instructed participants to point to one of the three sounds in the triplet. As in Strelnikov *et al*.^22^, in both training protocols audio-visual stimulation occurred in 75% of trials, whereas the remaining 15% consisted in auditory-only stimulation. Crucially, both groups were tested before and after training for their ability to localise a single auditory target delivered in front space. This *egocentric* sound localisation test was performed in both azimuth and elevation, because changes in performance following monaural plugging have been reported along the horizontal as well as vertical dimensions^30,31^. To assess training generalisation effects the stimulation used in pre- and post-training phases was a spoken syllable, instead of the white noise bursts used during training. Finally, a control group that did not perform any training was also included in the study to assess performance changes unrelated to training.

If allocentric and egocentric auditory spatial coding interact with one another, training benefits should transfer across *different* reference frames. This predicts that allocentric training should improve performance also in the subsequent egocentric task. Alternatively, partial segregation between allocentric and egocentric auditory spatial coding predicts that only the group that performed both training and test within the *same* reference frame (i.e., the egocentric training group) can improve performance.

## Material and Methods

### Participants

Forty-five normal hearing participants took part in the study and were assigned to one of three experimental groups: Allocentric Training Group (AlloT, N = 15, mean age = 23, *SD = *2.7, 10 females), Egocentric Training Group (EgoT, N = 15, mean age = 24, *SD = *3.9, 12 females), Control Group (Control, N = 15, mean age = 21, *SD = *2, 12 females). All participants reported no history of auditory or neurological disease and had normal or corrected to normal vision. Pure tone audiometry (250, 500, 1000, 2000, 4000 Hz) was used to screen participants for hearing loss, and served to assess the efficacy of the ear-plug in each participant (see Supplementary Materials and Fig. 1S). The study was conducted according to the research ethics regulation of the University of Trento and in line with the Declaration of Helsinki (1964, amended in 2013). Participants read and signed an informed consent before taking part in the experiment and received monetary reimbursement (7 €/hour) or course credits for participation.

### Apparatus

**Figure 2:**
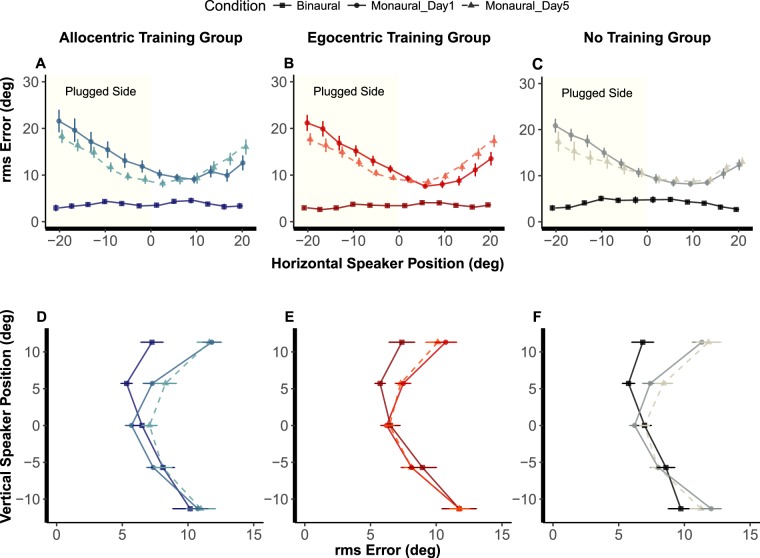
Performance in the sound localisation test. Rms errors for each listening condition (binaural, monaural day 1 and monaural day 5; for clarity performance on day 3 is not shown, see Table 1S and 2S for mean values) are displayed as a function of speaker position, separately for each group, for azimuth (**A**–**C**) and elevation (**D**–**F**). Thicker axes highlight horizontal speaker position (**A**–**C**) and vertical speaker position (**D**–**F**), respectively. Error bars show the standard error.

The experiment was administered in a reverberant room (4.2 × 5.0 m; mean background noise = 50 dB SPL). The experimental setup consisted in a custom-made rectangular wooden panel (95 × 60 cm) covering a visual angle of 43°, in which 60 round-shaped speakers (5 cm diameter of Mylar; Pro Signal ABS-210-RC range 350–20,000 Hz, 8K, 1W RMS Power) were mounted, arranged in a matrix of 5 rows and 12 columns (see Fig. 2S). A white acoustically transparent fabric was placed in the frontal part of the panel, to prevent vision of the sound-sources and to serve as screen for projecting all visual stimulations. Visual stimuli were projected using an LCD projector (LG HW300G; resolution: 1280 × 1024) connected to the stimulation PC (Dell Precision T3400). The panel was mounted on a wooden support and placed on a table 120 cm from participant’s head, with its centre approximately at ear level (Fig. 1A). The integrated sound card of the PC, connected to an external loudspeaker for sound amplification, was used for delivering all audio signals. Each signal was switched between 30 relays (NEC MR62-4.5 USB) by using the digital output of two National Instrument boards (NIusb-6259) to activate the desired speaker. Auditory stimulation was controlled using custom-made Matlab scripts (Mathworks R2015b, 32-bit).

### Procedure

The overall experimental protocol lasted five consecutive days (Fig. 1B). Hearing thresholds were measured by audiometric test for each participant on day 1 for right and left ear in normal hearing condition and left ear in plugged condition. In addition, hearing threshold of the plugged ear was assessed at the beginning of each testing day (see Supplementary Materials) to ensure that plugging was comparable across sessions.

On day 1, participants performed the test procedure in binaural (Binaural) and monaural hearing condition (Monaural day 1), in separate consecutive blocks. The same testing in monaural condition was repeated after two days (Monaural day 3) and on the last day (Monaural day 5). During the test procedure participants were administered a single-sound localisation task. We presented a stimulus (single spoken Italian syllable: counterbalanced number of occurrence between ‘BA’, ‘RO’ and ‘GU’, 500 ms duration) from a position of our stimulation setup and participants were required to localise the heard sound by moving a mouse cursor (hand icon, projected on the wide screen) to the location of the perceived sound, and validate their response with a left-click (Fig. 1C). In each testing phase we presented a total of 180 trials, 3 for each possible position in randomised order. The entire session lasted about 15 minutes and was divided into two blocks of 90 trials each.

Training occurred from day 2 to day 5 (four sessions of training in total), always with the left ear plugged (see Supplementary Materials). For the AlloT group, training was modelled on the auditory space bisection task proposed by Gori and colleagues^29^. Three consecutive sounds (white noise bursts, 500 ms each) were presented at different horizontal positions, at identical elevation. In each trial, sounds originated from three positions with same elevation but different azimuth. The first sound of the triplet was delivered with equal probability from the speaker located at −20°, −17° or −13° to the left of the participant, whereas the third sound was always delivered 9 speakers to the right of the first (e.g., first sound at −20°, third sound at 13°). Critically, the second sound was delivered at one of the intermediate positions between the first and the third, from immediately adjacent to the first to immediately adjacent to the third. In each trial, participants had to indicate whether the second sound, was closer to the first or to the third one. Responses were collected using the computer keyboard. Our choice to change the position of the first sound (unlike the classic procedure by Gori and collaborators^29^) aimed to reduce the possibility that participants solved the allocentric task through egocentric strategies^32^, paying attention exclusively to the second sound while assuming that the first and third remained at identical positions throughout the experiment. Elevation of sound triplets changed unpredictably across trials (5 possible elevation levels), with each elevation sampled with equal probability within each block. The session was divided in 4 blocks of 60 trials each (total of 240 trials), for an overall duration of about 25 minutes.

In 75% of trials, a white dot (10 pixel diameter) appeared at the time of the auditory stimulation, indicating exactly the location of source location. To emphasise the spatial relation between the sounds a red line (2 pixels width) was presented to connect consecutive sounds (Fig. 1D). In the remaining 25% of trials, sounds were presented without visual stimulation to prevent participants from solving the task only relying on visual stimulation. The percentage of audio-visual stimulation was based on the work of Strelnikov and colleagues^22^.

For the EgoT group, in each trial participants listened to three consecutive sounds (white noise bursts, 500 ms each) exactly as detailed above for the AlloT group. However, after presentation of the last sound (third) a visual instruction appeared on screen indicating which of the three sounds localise (e.g. ‘S1’, to indicate the first sound). Participants were required to point to target position using the mouse cursor that appeared on the white panel only at the moment of the response. As in the allocentric training, in 75% of trials a white dot indicated the position of each sound (Fig. 1E). No red lines appeared connecting the dots. In the remaining 25% of trials, sounds were presented without visual stimulation. Again, participants received a total of 240 trials per session, divided in 4 blocks of 60 trials each. A single training session lasted approximately 25 minutes.

Three methodological aspects are worth noting. First, the auditory stimulation in both training procedures was completely identical, and the difference between training procedures concerned only the visual stimulation and the task. Although each individual sound likely underwent an initial egocentric coding in both training procedures, critical to the allocentric training task was the spatial comparison between sounds, which entails an allocentric spatial representation. Second, to observe potential generalisation effects of the training paradigms we employed different stimuli and tasks between training and testing sessions, in line previous works^33–35^. Third, unlike most of the previous works^18^, we introduced in the design a control group that did not take part in any training procedure (No Training group, NoT), but performed only testing of sound localisation at day 1, day 3 and day 5.

### Data analysis

Our stimulation setup allowed the presentation of auditory stimuli both in azimuth and elevation, therefore we have been able to observe different degrees of performance in the two dimensions (see Tables 1S and 2S for cumulative error measurements in all conditions). In the present work, we investigated performance through rms error and signed error, following Hartmann^36^ (see also refs^6,37^). The rms error represents the root mean squared difference between speaker position and subject’s response. The Signed error represents mean difference between response and correct sound-source position and it could be either positive or negative. When considering azimuth, positive values indicate a rightward bias; when considering elevation, positive values indicate an upward bias. We calculated each error separately for each response component: azimuth and elevation. In case of sphericity assumption violations we adopted the Greenhouse-Geisser method of correction.

## Results

Figure 2 shows rms error at the beginning of the protocol for both binaural and monaural listening conditions (day 1, shown with bold lines) and at the end of the protocol (day 5, shown with dashed lines). Performance is presented separately for each of the three groups as a function of horizontal speaker position (Fig. 2A–C) and vertical speaker position (Fig. 2D,F). In the following sections we describe (1) the ear-plug effects on sound localisation on day 1; (2) performance changes at day 5; (3) performance changes at day 3.

### Ear-plug effects on sound localisation on day 1

To examine sound localisation performance on day 1, we entered rms errors in a mixed Analysis of Variance (ANOVA) with listening condition (binaural, monaural), stimulation side with respect to the plugged ear (ipsilateral, contralateral), and stimulation eccentricity with respect to listener’s body midline (2°, 6°, 9°, 13°, 17°, 20°) as within-participant variables, and group (AlloT, EgoT and NoT) as between-participants variable.

**Figure 3:**
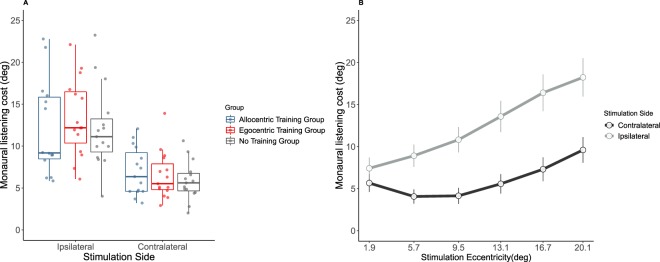
(**A**) Monaural listening cost in the horizontal plane as a function of Stimulation Side (Ipsilateral/Contralateral to the plug) and experimental group. (**B**) Monaural listening cost in the horizontal plane, irrespective of group, as a function of Stimulation Eccentricity and Stimulation Side. Error bars represent confidence intervals of the mean.

This analysis revealed a 2-way interaction between listening condition and stimulation side (F(1,42) = 44.57, p < 0.001, η^2^ = 0.69) and a significant 3-way interaction between listening condition, stimulation side and stimulation eccentricity (F(1.87,78.46) = 16.69, p < 0.001, η^2^ = 0.28). None of these interactions was further modulated by group (all F-values < 1). To study these interactions, we calculated the cost of monaural compared to binaural listening (from now on termed *‘monaural listening cost’*) as the difference in rms error between the two conditions (positive values indicate worse performance in monaural listening). We found that monaural listening cost was overall larger for sounds delivered on the side ipsilateral to the plug (12.4° ± 5.4) compared to the contralateral side (5.9° ± 2.6; see Fig. 3A). Interestingly, monaural listening cost varied substantially between-participants: some participants were minimally affected by the ear plug, whereas others localised all sounds towards the open ear. Finally, as revealed by the 3-way interaction reported above, monaural listening cost increased as a function of eccentricity, particularly on the side ipsilateral to the plug (see Fig. 3B). The omnibus ANOVA also revealed the main effects of listening condition, stimulation side and stimulation eccentricity (all F-values > 30.00), as well as the two-way interactions stimulation side* stimulation eccentricity and listening condition*stimulation eccentricity (all F-values > 14.00), which were subsidiary to the higher order interactions described above.

A similar ANOVA on mean signed error was run to examine systematic biases in sound localisation. This analysis revealed again a 3-way interaction between listening condition, stimulation side and stimulation eccentricity (F(1.87,78.46) = 16.69, p < 0.001, η^2^ = 0.28), which was not modulated by group (F(3.74,78.46) = 0.48, p = 0.74, η^2^ = 0.02). To study this interaction, we calculated the bias induced by monaural compared to binaural listening (from now on termed ‘*monaural listening bias*’) as the difference in signed error between the two conditions (positive values indicate a bias towards the side contralateral to the plug). This bias is shown in Supplementary Fig. 3S as a function of eccentricity. It emerged as strong bias to respond towards the hearing side for sounds originating from the space ipsilateral to the plug (average bias: 13.58 ± 8.03; for all eccentricities t-test comparisons against zero p < 0.001). Interestingly, we also found a mild bias towards the body midline when responding to most eccentric sounds contralateral to the plug (−5.31 ± 4.66, −6.99 ± 5.13, −8.39 ± 5.04, for the three most eccentric positions, respectively; all t-test comparisons against zero p < 0.001).

In the vertical plane, sound localisation in binaural listening was overall less precise compared to localisation in azimuth (rms error: azimuth = 3.7° ± 1.7; elevation = 7.6° ± 3.2; paired t-test, t(44) = 12.47, p < 0.001, Cohen’s d = 1.86). To study whether alteration of binaural cues impacted also on sound elevation perception we entered rms error along the vertical dimension in a mixed ANOVA with listening condition (binaural, monaural), vertical speaker position (−11°, −6°, 0°, 6°, 11°) as within-participants factors, and group (AlloT, EgoT and NoT) as between-participants factor. This analysis revealed that rms error increased from binaural to monaural listening (8.3° ± 2.0, 9.3° ± 1.1, respectively; F(1,42) = 14.33, p < 0.001, η^2^ = 0.25). Localisation errors increased particularly for sounds above ear level ( + 5°: 5.6° ± 1.7 vs. 7.4° ± 2.3; + 11°: 7.1° ± 3.4 vs. 11.3° ± 2.9), resulting in a significant two-way interaction between listening condition and vertical speaker position (F(1.46, 61.33) = 15.95, p < 0.001, η^2^ = 0.27). As for the analysis on the horizontal dimension, performance decrement determined by monaural plugging was comparable across groups (no main effect or interaction involving the group factor emerged; all F values < 1).

A similar ANOVA on Signed Error in the vertical dimension, revealed an interaction between listening condition and vertical speaker position (F(2.15, 90.31) = 55.98, p < 0.001, η^2^ = 0.54). Sounds from above the horizontal midline were localised downward in binaural listening (−2.9° ± 3.7) and even more so in monaural listening (−7.3° ± 3.0; t(44) = 7.36, p < 0.001, Cohen’s d = 1.1). By contrast, sounds from below the midline were localised upward in both binaural (7.7° ± 4.6) and monaural (7.7° ± 3.3) listening conditions alike (t(44) = 0.07, p = 0.942).

### Change in performance at day 5

Changes in sound localisation performance in azimuth and elevation between day 1 and day 5 are shown in Fig. 2, separately for the trained groups and the no-training group (compare solid and dashed lines depicting monaural listening conditions at day 1 and day 5, respectively).

To study the difference in monaural listening performance between the first and the last testing day, we entered rms errors in a mixed Analysis of Variance (ANOVA) with day (day 1, day 5), stimulation side, stimulation eccentricity and group as before. This analysis revealed a significant 2-way interaction between day and stimulation side (F(1,42) = 23.82, p < 0.001, η^2^ = 0.36), as well as a 3-way interaction between day, stimulation side and stimulation eccentricity (F(2.81,118.10) = 9.56, p < 0.001, η^2^ = 0.18). None of the above interactions was further modulated as a function of group (all F-values < 1).

**Figure 4:**
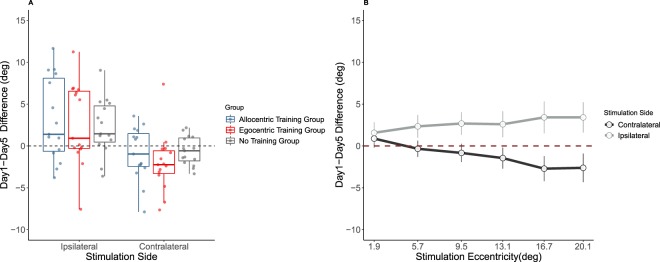
(**A**) Day1-Day5 Difference in the horizontal plane as a function of Stimulation Side (Ipsilateral/Contralateral to the plug) and experimental group. (**B**) Day1-Day5 Difference in the horizontal plane irrespective of group as a function of Stimulation Eccentricity and Stimulation Side. Error bars represent confidence intervals of the mean. Positive values indicate performance improvement, negative values performance decrement.

To study these interactions, we calculated performance change as the difference in rms error between day 1 and day 5 (from now on termed ‘*Day1-Day5 Difference*’). On the side ipsilateral to plug, the rms error was smaller on day 5 (13.54 ± 4.18) compared to day 1 (16.21 ± 5.31; t-test: t(44) = 4.10, p < 0.001, Cohen’s d = 0.61, corrected for multiple comparison; Day1-Day5 Difference = 2.67 ± 4.37). By contrast, on the side contralateral to the plug, the rms error significantly increased from day 1 (9.87 ± 2.44) to day 5 (11.05 ± 2.73; t-test: t(44) = 2.73, p = 0.02, Cohen’s d = 0.41, corrected for multiple comparisons using Bonferroni; Day1-Day5 Difference = −1.18 ± 2.89; see Fig. 4A). Day1-Day5 Difference also changed as a function of eccentricity on both sides, but particularly on the side ipsilateral to the plug (see Fig. 4B). The omnibus ANOVA revealed also the significant main effects of stimulation side and stimulation eccentricity (all F-values > 30.00) and the two-way interaction stimulation side* stimulation eccentricity (F(1.83, 76.73) = 12.56, p < 0.001, η^2^ = 0.23), both subsidiary to the higher order interactions described above.

A similar ANOVA performed on mean signed error was conducted to study if any systematic bias changed between day 1 and day 5. This analysis revealed the significant main effects of day (F(1,42) = 25.5, p < 0.001, η^2^ = 0.375), stimulation side (F(1,42) = 673.21, p < 0.001, η^2^ = 0.94), stimulation eccentricity (F(4.05,170.25) = 12.49, p < 0.001, η^2^ = 0.22), and the two-way interaction side*eccentricity (F(1.85,170.25) = 77.74, p < 0.001, η^2^ = 0.92). No higher order interaction emerged (all F-values < 1), as well as no main effect or interactions involving the between participants factor group (all F-values < 1.1).

Overall, signed error changed between day 1 and day 5 (5.24 ± 5.37, 2.06 ± 4.09, respectively, t-test: t(44) = 5.14, p < 0.001, Cohen’s d = 0.77). Similar to monaural listening at day 1, results on monaural listening at day 5 confirmed localisation bias toward the hearing side for sounds ipsilateral to the plug (9.99 ± 7.21), and a bias towards the midline for sounds originating from the space contralateral to the plug (−5.89 ± 7.12).

Interestingly, sound localisation performance in the vertical plane remained unchanged between day 1 and 5 (see lower panels in Fig. 2). A mixed ANOVA on rms error, with day (day 1, day 5) and vertical speaker position (−11°, −6°, 0°, 6°, 11°) as within-participants factors, group (Allot, EgoT and NoT) as between-participants factor, revealed no significant main effect of day (F(1.527,61.081) = 1.54, p = 0.224, η^2^ = 0.04) or group (F(2,40) = 0.39, p = 0.68, η^2^ = 0.02), nor any two-way or three way interactions (all F-values < 1.4).

### Change in performance at day 3

Having established that monaural listening performance in rms error changed between the first and the last day of our protocol, we investigated whether this dependent variable changed even after two days, namely at day 3. To this aim, we entered azimuthal rms errors in a mixed Analysis of Variance (ANOVA) with monaural testing day (day1, day 3), stimulation side with respect to the plugged ear (ipsilateral, contralateral), and stimulation eccentricity with respect to listener’s body midline (2°, 6°, 9°, 13°, 17°, 20°) as within-participant variables, and group (AlloT, EgoT and NoT) as between-participants variable (see Tables 1S and 2S for mean values).

This analysis revealed a 2-way interaction between day and stimulation side (F(1,40) = 12.18, p = 0.001, η^2^ = 0.23) and the 3-way interaction day*stimulation side*stimulation eccentricity (F(2.58,103.26) = 6.53, p < 0.001, η^2^ = 0.14). None of these interactions were modulated by group (all F-values < 1 s). To study these interactions, we calculated localisation performance difference in the horizontal plane between the first (day 1) and the third (day 3) testing day in monaural listening as the difference in rms error between the two testing days (from now on termed ‘*Day1-Day3 Difference*’). On the ipsilateral side, rms error reduced on day 3 (13.27 ± 4.54) compared to day 1 (16.21 ± 5.31; t-test: t(42) = 4.18, p < 0.001, Cohen’s d = 0.64, corrected for multiple comparisons using Bonferroni; Day1-Day3 Difference = 2.50 ± 3.92). On the side contralateral to the plug, however, no change in rms error emerged from day 1 (9.87 ± 2.44) to day 3 (10.32 ± 2.38) (t-test: t(42) = 1.03, p = 0.62, Cohen’s d = 0.16, corrected for multiple comparison; Day1-Day3 Difference = 0.03 ± 2.24). Day1-Day3 Difference increased as a function of eccentricity in the plugged side, whereas in the unplugged side reached significance only for one eccentricity (see Supplemetary Fig. 4S). The main effects of day, stimulation side and stimulation eccentricity (all F-values > 10.00) as well as the two-way interactions stimulation side* stimulation eccentricity (F(1.84, 73.57) = 13.50, p < 0.001, η^2^ = 0.25) reached significance, but were subsidiary to the higher order interactions described above.

In the vertical plane localisation performance did not change between day 1 and day 3. *Rms error* in the vertical plane was studied using an ANOVA with day (day 1, day 3) and vertical speaker position (−11°, −6°, 0°, 6°, 11°) as within-participant factor, and group (Allot, EgoT and NoT) as between-participants factor. The analysis revealed no significant main effects or interaction involving the group variable (all F-values < 1.5).

### Follow-up analyses

**Figure 5:**
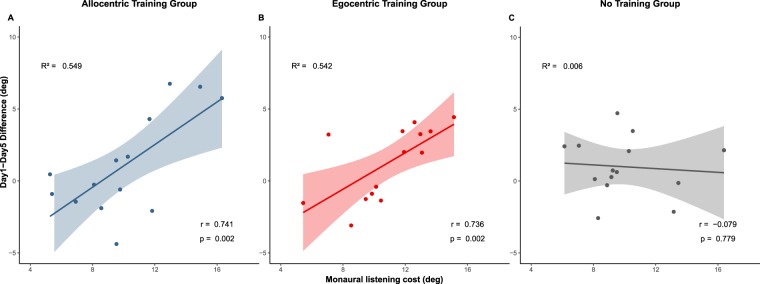
(**A**–**C**) Relation between monaural listening cost and Day1-Day5 Difference as a function of group. The trained groups (AlloT and EgoT, panels A and B, respectively) show a positive and significant correlation between monaural listening cost at day 1 and the performance difference between day 1 and day 5. No such relation emerged for the No Training group (**C**).

The first follow-up analysis stem from the observation that monaural listening cost in horizontal dimension at day 1 was substantially different across participants (Fig. 3A). We asked whether such variability at day 1 could predict performance changes observed at the end of our protocol (day 5). To this aim, we analysed the relation between *monaural listening cost* at day1 and *Day1-Day5 Difference* using Pearson correlations. Correlation plots are shown in Fig. 5, separately for the three groups. In both training groups a significant relation between monaural listening cost and Day1-Day5 Difference emerged: the more participants decreased their performance in azimuth due to monaural plugging on day 1, the more they changed localisation performance after training (AlloT Group: r = 0.74, p = 0.002, EgoT Group: r = 0.74, p = 0.002). By contrast, Day1-Day5 Difference was unrelated to monaural listening cost in the No Training group (r = −0.079, p = 0.78). An ANCOVA with day1-day5 difference as dependent variable, group (AlloT, EgoT and NoT) as independent variable and monaural listening cost as covariate, showed a significant 2-way interaction between group and monaural listening cost (F(2,39) = 5.69, p = 0.007, η^2^ = 0.14).

The second follow-up analysis stem from the observation that Day1-Day5 Difference on horizontal plane was positive on the plugged side (suggesting performance improvement), but negative on the unplugged side (suggesting performance decrement). One interpretation for this unexpected finding is that recalibration of auditory space between day 1 and 5 emerged as overall shift of responses towards the unplugged side – rather than a re-learning of auditory-cue space correspondences. If this is the case, Day1-Day5 Difference on the plugged side should correlate with Day1-Day5 Difference in the unplugged side across participants. We tested this hypothesis using correlations (Pearson) and found no relation between ipsilateral improvement and contralateral decrease, neither when treating all participants together (r = 0.03, p = 0.86) nor when splitting them as a function of group (AlloT: r = 0.27, p = 0.34; EgoT: r = −0.15, p = 0.58; NoT: r = −0.07, p = 0.080).

## Discussion

In the present study, we examined the relation between allocentric and egocentric reference frames in spatial hearing, in the context of multisensory guided training of sound localisation. First, we confirmed that the method adopted for auditory cues alteration (monaural ear plugging) can effectively impair sound localisation abilities in the horizontal dimension. In addition, we extended this classic finding by showing that sound localisation impairments emerged also in the vertical dimension, and by documenting substantial inter-participants variability in the monaural listening cost determined by the ear-plug. Second, after four days of protocol (day 5), we measured performance improvements on single sound localisation on the side ipsilateral to the plug (left), which emerged together with concomitant performance decrements on contralateral space (right). Notably, these performance changes emerged only in the horizontal dimension, whereas monaural sound localisation in the vertical dimension remained entirely unaffected. Third, we found that performance changes occurred with comparable strength in all tested groups – i.e., irrespective of whether participants were trained or untrained. From a theoretical perspective, this finding implies that listeners can change their sound localisation performance even in absence of error feedback signal. From a methodological point of view, it stresses the importance of including a no training group when assessing the efficacy of sound localisation training methods. Finally, we proposed a new audio-visual protocol based on allocentric coding of sound, extending the principles of multisensory stimulation previously implemented only using egocentric procedures. In the following sections, we will discuss each of these novel contribution in details.

In agreement with previous findings we show performance decrement from binaural to monaural listening (ear-plugging), mostly in the space ipsilateral to the plug. However, the impact of monaural plugging on sound localisation performance was markedly different between participants. Inter-individual variability of the consequences of ear plugging has been reported previously^38^, but its origin remains to be ascertained. It could reflect inter-individual efficacy of the plug, different sensitivity to auditory cues alteration, or a combination of the two. An interesting finding of the present study is the impact of this inter-individual variability of the plug-effect (which we termed ‘monaural listening cost’) on performance change after four days of training (which we termed ‘Day1-Day5 Difference’). Although sample size of each experimental group was limited, it is noteworthy that in our two trained groups the magnitude of training effects was linearly related with the amount of sound localisation deficit induced by the plug (see Fig. 5A–C). Participants who were more affected by the ear plug changed their performance post-training to a greater extent, compared to participants who were minimally affected by monaural occlusion. Intriguingly, this positive relation was completely absent in the control group – which just repeated the test after 2 and 4 days. Given the limited number of participants in each group (N = 15), these correlation results should be taken with caution. Yet, positive relations between earplug effect and training benefit have previously been reported in humans with similar sample sizes^18^ (but see ref.^39^ for contrasting results). In particular, in a study on spatial hearing re-learning after monaural ear-moulding, Van Wanrooij and Van Opstal^18^ showed that participants whose spectral cues were disrupted the most achieved better post-training results (N = 11), and learned at faster rates compared to participants who were minimally affected by monaural ear-moulding. The functional model proposed by Van Opstal (2016) assumes that a new correspondence between auditory cues and space is stored whenever a discrepancy is detected between the spatial outcome of an existing sound-space correspondence (SSC) and the actual stimulus position, leading to the consolidation of new SSC with repeated exposures. In this context, the observed relation between monaural listening cost and Day-Day5 Difference might be interpreted as the consequence of the perceived discrepancy between the response and the actual sound position. This discrepancy was only experienced during training and was clearly largest for those participants who were maximally affected by the plug. Indeed, no such relation between ear-plug effect and change in performance between day 1 and day 5 was detected in the no-training group (which never received feedback).

This finding suggests that qualitative differences might exist between the trained groups and the no-training group, despite the quantitatively comparable performance. Performance changes in individuals with altered auditory cues who received no error-feedback signal has been recently documented using other methods. Zonooz and colleagues^40^ tested sound localisation in the vertical plane using spectrally poor (band limited) sounds in trained vs. untrained participants. While performance improvements were larger in participants trained with visual feedback, even those participants who did not received performance-related feedback showed sound-localisation improvements in vertical plane. Performance changes in the no-training group are compatible with the theoretical framework provided by Keating and King^15^. The authors proposed that adaptation to altered sound localisation cues can result from two different (though not mutually exclusive) mechanisms: cue-reweighting and cue-remapping. Cue-reweighting is a fast adaptation process, in which the relative contribution (i.e., weight) of the available auditory cues is changed to accommodate the intervening modulations (e.g., ear-plugging, as here). Instead, cue-remapping is a slowly emerging adaptation phenomenon in which a new correspondence between available auditory cues and space is progressively encoded. The observed differences between trained and un-trained participants may relate to these two different adaptation mechanisms. Specifically, the no-training group may have primarily changed its performance exploiting a fast cue-reweighting mechanism. By contrast, the sensory feedback provided in the audio-visual trainings may have triggered a slower process of sensory recalibration, in which new sound-space correspondences started to emerge from a combination of cue-reweighting and cue-remapping processes. This suggests that longer and more consistent training procedure may be essential to achieve proper cue-relearning. In this sense, it is possible that our short training (25 minutes per sessions, for 4 consecutive days) captured only the intermediate phase of the entire adaptation process, in which cue-reweighting – but not a full and stable cue-remapping mechanism – has been accomplished. This could also partially explain the combination of ipsilateral improvement and contralateral decrement measured in the trained group.

The present study also introduced a novel training approach based on audio-visual coding of sounds in *allocentric* coordinates. Previous research exploring the effects of training in the context of reversibly altered auditory cues almost exclusively adopted an egocentric frame of reference (for a review see ref.^16^). One notable exception is a study conducted by Gori and colleagues^41^, conducted with blindfolded hearing participants tested in binaural listening. The procedure entailed an auditory spatial bisection task similar to the one adopted here, as well as training sessions aimed at improving allocentric coding using multisensory feedback. Specifically, the experimental set-up comprised nine speakers, each associated with one vibrotactile stimulator placed on the forearm of the participant. Speakers and vibrotactile stimulators were kept spatially aligned throughout the task. The tactile feedback group listened to triplets of acoustic stimuli (as in the present work), paired with delayed (200 ms) spatially-congruent tactile stimulation. During training, participants were required to attend the multisensory stimulation but performed a different task, which consisted in detecting an occasional higher tone. Performance in the auditory bisection task improved in the tactile feedback group, but not in participants who received only a verbal feedback about sound position, or participants who received no feedback.

While this audio-tactile study highlights the potentials of multisensory stimulation in promoting allocentric auditory space perception, two differences with the current work are noteworthy. First participants were not explicitly required to perform an allocentric coding of sounds during training, unlike our audio-visual allocentric task. Second, in Gori *et al*.^41^ stimuli and task remained identical throughout the experiment, limiting our understanding of generalisation of training effects. Conversely, our study introduces, for the first time, an allocentric training procedure based on *relative* position of multiple sounds for the purpose of changing auditory space mapping. Importantly, the structure of our paradigm, with differences between training and test sessions both in terms of stimulation (spoken syllables, rather than white noise bursts) and task (localisation of a single stimulus, rather than egocentric/allocentric processing of triplets of sounds) allows assessing learning generalisation effects. Generalisation effects have strong implications for translational application of training procedures to applied or clinical settings, and are thus considered a key requirement when assessing the efficacy of training procedures^33,34,42,43^. Our novel allocentric training procedure also extends to allocentric coding the multisensory training principles^44–47^ for the purpose of acoustic space re-learning. We provided participants a visual counterpart not only of the position of the sounds, but also of the spatial relationship between them. The rationale for adding lines between the dots (Fig. 1D) was to emphasise visuo-spatial encoding of the acoustic scene. In addition, we reasoned that they could promote the use of visuo-spatial mental imagery^48^ during the response phase, in agreement with the evidence that vision can facilitate allocentric representations of space^49^. We acknowledge, however, that the effectiveness of the added lines connecting the dots should not be taken for granted, as a proper control condition is missing in the present paradigm.

In conclusion, our work constitutes a first attempt to address a largely unexplored aspect of sound localisation abilities – namely the interaction between egocentric and allocentric representations. Addressing this domain is important because it could potentially introduce a novel approach to auditory spatial training. In vision, studies on egocentric vs. allocentric space coding have shown partial dissociation^50–55^ as well as interactions^56–60^ between these representations. Likewise, integration and interaction between different reference frames has been proposed to be necessary for an efficient tactile localisation^61^. To the best of our knowledge, our study provides the first investigation aimed at assessing such interaction between reference frames in the auditory domain. It has been recently proposed that egocentric and allocentric representations may be linked in a hierarchical manner, with egocentric processing involving only a subpart of the wider neural network subtending allocentric processing^62^. If this is the case, exploiting allocentric training could foster space re-learning to a greater extent and prove more effective compared to training based on egocentric space representations. Testing this hypothesis was beyond the scopes of the present work, and it implies testing the efficacy of egocentric and allocentric training on sound localisation tests conducted on both reference frames (i.e., egocentric and allocentric). Yet, this follow-up study could exploit the current findings to further understand the contribution of allocentric auditory space processing in sound localisation re-learning.

## Supplementary information

Supplementary Information

## Data Availability

A public data repository is available at the following link: https://osf.io/rczbg/.
