# Supplementary material for: Interactions between egocentric and allocentric spatial coding of sounds revealed by a multisensory learning paradigm
Source: Sci Rep. 2019 May 27;9:7892. doi: 10.1038/s41598-019-44267-3 (PMC6536515; doi:10.1038/s41598-019-44267-3)
Supplement: Supplementary file 1 — Supplementary Information [file 41598_2019_44267_MOESM1_ESM.pdf]

Supplementary Information

Interactions between egocentric and allocentric spatial coding of sounds revealed by a multisensory learning paradigm

Giuseppe Rabini[a], Elena Altobelli[b], Francesco Pavani[a,b,c]

a. *Centre for Mind/Brain Sciences (CIMeC), University of Trento*
b. *Department of Psychology and Cognitive Sciences (DiPSCo), University of Trento*
c. *IMPACT, Centre de Recherche en Neuroscience Lyon (CRNL), France*

Giuseppe Rabini, MS.D. *
CIMeC – Centre for Mind/Brain Science, University of Trento
Via Angelo Bettini 31, 38068, Rovereto (TN), Italy
Phone: +39 0464808726
giuseppe.rabini@unitn.it

Elena Altobelli, MS.D.
Department of Psychology and Cognitive Science, University of Trento
Via Angelo Bettini 84, 38068, Rovereto (TN), Italy
Phone: +39 3488091454
elena.altobelli@alumni.unitn.it

Francesco Pavani, Ph.D.
CIMeC – Centre for Mind/Brain Science, University of Trento
Via Angelo Bettini 31, 38068, Rovereto (TN), Italy
Phone: +39 0464808674
francesco.pavani@unitn.it

*Corresponding Autho

Supplementary material and methods

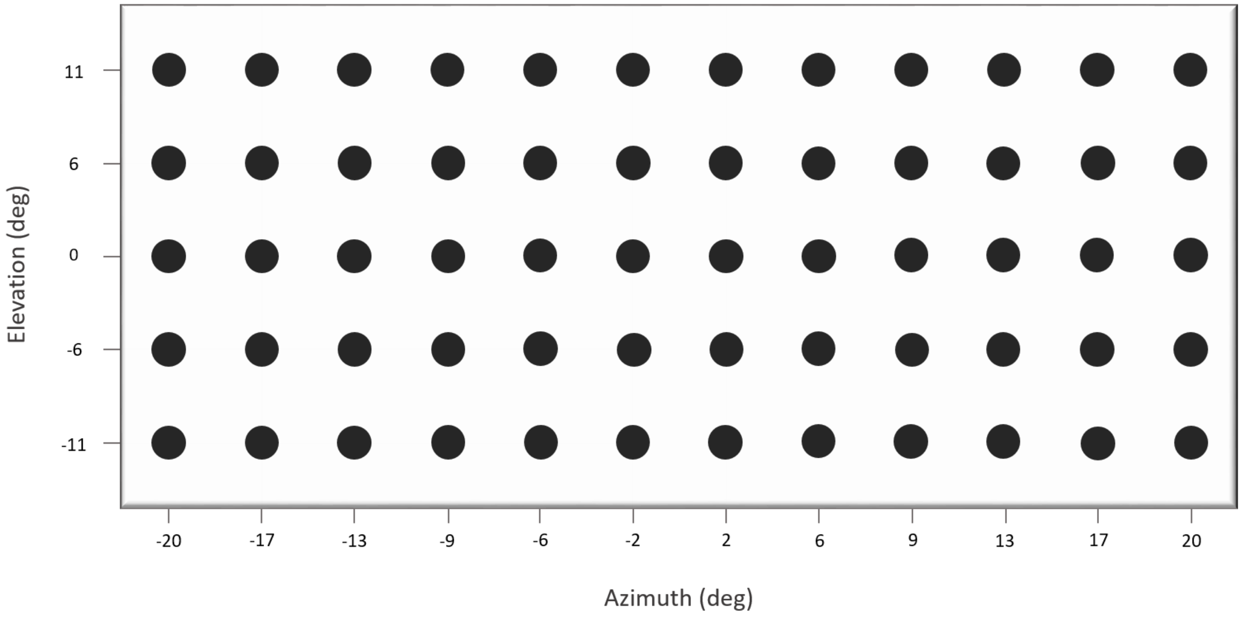

Figure 1S. Schematic representation of the stimulation setup. A matrix of speakers, arranged in 5 rows and 12 columns, were fixed on a rectangular wooden panel, allowing sound presentation both in azimuth and elevation. Black circles represent auditory stimulation speakers with specific coordinates in horizontal and vertical plane. Visual stimuli were then projected on the white acoustically transparent fabric covering the speakers.

Ear plugging method and audiometric testing

We used EAR Classic foam earplugs (3M PP 01 002; mean attenuation values as reported by manufacturing data: 30 dB, 24 dB, 22 dB for high, medium, low frequencies respectively; Single Number Rating = 32 dB) to simulate a unilateral conductive hearing loss and therefore decreasing spatial hearing abilities. The plug was always applied to the left ear. An intermitted approach (see Ref [13] in main manuscript) was adopted, applying the plug at the beginning of each testing session and removing it at the end. Hearing threshold at the plugged ear was measured before each training session by audiometric test (Grason Stadler GSI 17 Audiometer) to quantify the plug-induced decrease of hearing threshold in each testing day. In the present experiment, mean attenuation value induced by the plug was 22.18 dB. Specifically, mean attenuation varied across frequencies tested (250 Hz: 14.52 dB; 500 Hz: 14.25 dB; 1000 Hz:18.89 dB; 2000 Hz: 29.64 dB; 4000 Hz: 33.61 dB; see also Fig. 2S). The experimenter explained the correct procedure for ear plugging (verbal instruction: “Roll the Ear Plug into a narrow tube. Put it in it, and hold it place for 20s with your fingertips”) to each participant at the beginning of each experimental session to ensure an optimal fitting of the plug. During experiment, plugged ear was further covered with an ear muff (3M 1445, mean attenuation values of 32 dB, 29 dB, 23 dB for high, medium, low frequencies respectively; Single Number Rating = 28 dB, as reported by manufactory data), while the corresponding opposite muff was removed.

Figure 2S shows mean audiometric values for unplugged ears at day 1 (left and right) and plugged ear in all training and testing days (left), for each frequency tested (250, 500, 1000, 2000, 4000 Hz). Differently from common practice, we measured hearing threshold in the same reverberant room in which experiment was later administered.

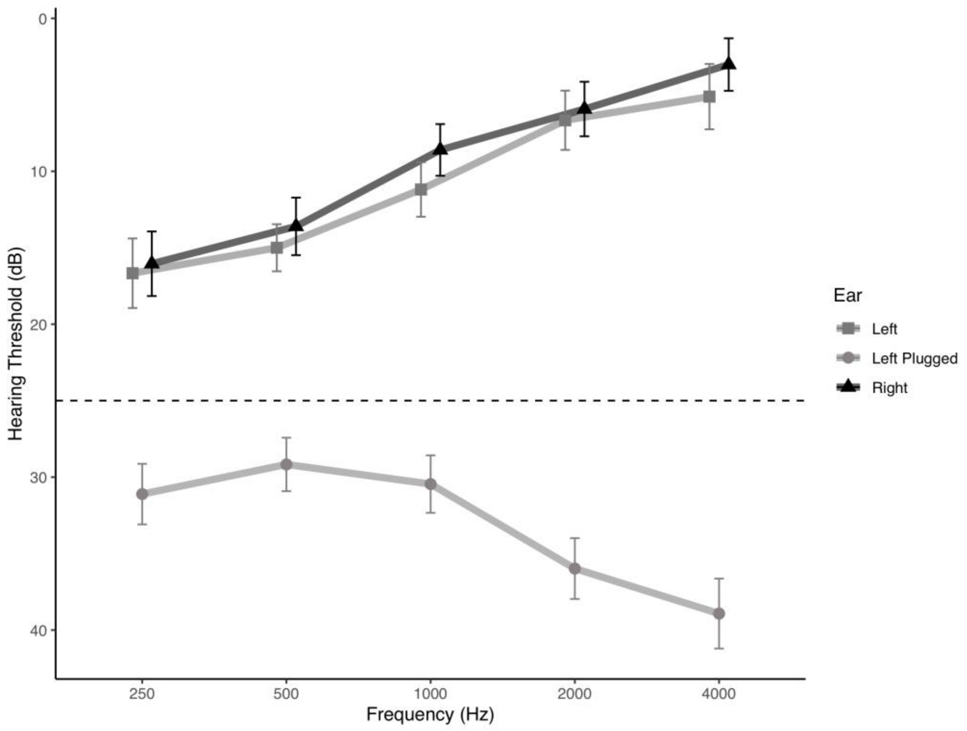

Figure 2S. **Audiometric Test**. Mean hearing threshold value for each frequency tested has been reported for right ear, left ear and left ear with the plug. For each frequency, hearing threshold of the plugged ear was clearly below 20 dB for left and right ear unplugged, and above 20 dB for left plugged ear. Error bars represent confidence intervals.

Supplementary Results

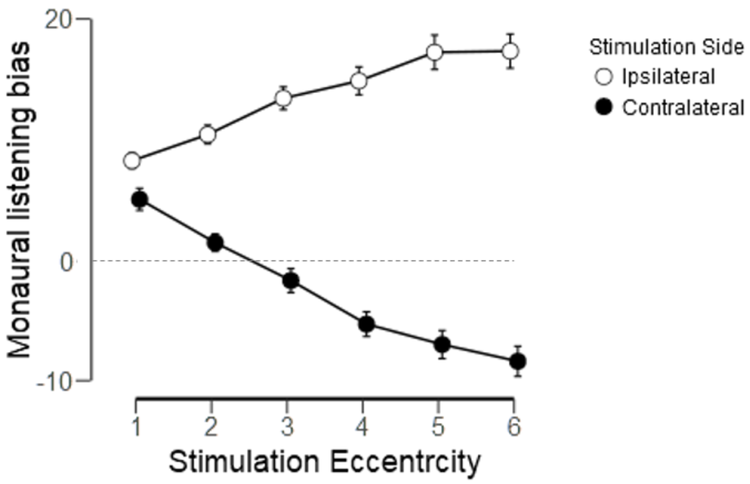

Figure 3S. Monaural listening bias as a function of stimulation eccentricity, divided by side of stimulation. Monaural listening bias represents the difference between signed error in monaural and binaural listening at day 1. Error bars show confidence intervals of the mean.

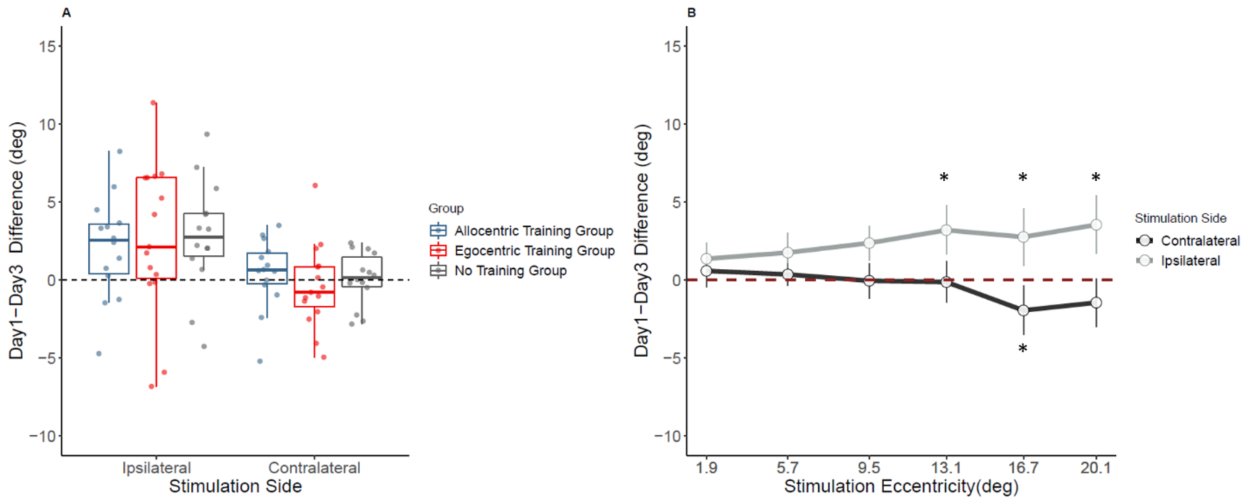

Figure 4S. (A) Day1-Day3 Difference in horizontal plane as a function of Stimulation Side (Ipsilateral/Contralateral to the plug). Day1-Day3 Difference, calculated as the difference between rms error in monaural listening at day 1 and day 3, is shown separately for each experimental group. **(B)** Day1-Day3 Difference in horizontal plane is shown as a mean of all groups, as a function of Stimulation Eccentricity and divided by Stimulation Side. Error bars represent confidence intervals of the mean.

Table 1S

Sound localisation performance in horizontal plane. Mean values (± SD) of rms Error and Signed Constant error in all experimental groups (Allocentric Training, Egocentric Training, No Training) in all listening conditions (binaural, monaural at day 1, day 3 and day 5).

	**Allocentric Training Group** (N = 15)				**Egocentric Training Group** (N = 15)				**No Training Group** (N = 15)			
Error Measure	***Binaural***	***Monaural***			***Binaural***	***Monaural***			***Binaural***	***Monaural***		
		Day1	***Day3***	***Day5***		***Day1***	***Day3***	***Day5***		***Day1***	***Day3***	***Day5***
rms error D	3,901 (1,573)	14,690 (4,151)	12,909 (2,564)	13,210 (2,650)	3,573 (0,375)	14,12 (2,931)	12,877 (2,997)	13,407 (2,302)	4,262 (1,508)	13,781 (2,405)	12,011 (3,048)	12,779 (3,071)
Signed constant error E	- 0,184 (1,101)	5,276 (7,015)	2,018 (4,147)	1,693 (4,269)	-0,531 (0,698)	4,973 (4,582)	2,429 (3,259)	1,590 (2,314)	0,031 (1,270)	5,462 (4,489)	2,961 4,383)	2,906 (5,275)

Table 2S

Sound localisation performance in vertical plane. Mean values (± SD) of rms error and Signed constant error in all experimental groups (Allocentric Training, Egocentric Training, No Training) in all listening conditions (binaural, monaural at day 1, day 3 and day 5).

	**Allocentric Training Group** (N = 15)				**Egocentric Training Group** (N = 15)				**No Training Group** (N = 15)			
Error Measure	***Binaural***	***Monaural***			***Binaural***	***Monaural***			***Binaural***	***Monaural***		
		Day1	***Day3***	***Day5***		***Day1***	***Day3***	***Day5***		***Day1***	***Day3***	***Day5***
rms error D	8,020 (2,265)	9,140 (0,992)	9,648 (1,639)	9,771 (1,413)	8,801 (2,254)	9,438 (1,184)	9,518 (1,016)	9,214 (1,070)	8,021 (1,507)	9,496 (1,246)	9,626 (0,837)	9,810 (0,962)
Signed constant error E	2,002 (3,718)	-0,328 (2,588)	-0,325 (4,196)	-0,335 (4,050)	2,959 (4,492)	0,634 (3,665)	1,295 (3,662)	0,686 (3,001)	2,518 (3,73)	0,279 (2,962)	-0,408 (3,603)	-0,242 (3,458)

Table 3S

Mean values (± sd) of Monaural listening cost, Day1-Day5 Difference and Day1-Day3 Difference computed on rms error, reported as a function of Stimulation Side (Ipsilateral/Contralateral to the plug) and Stimulation Eccentricity.

Dependent Variable	Stimulation Side	Stimulation Eccentricity					
		**2°**	**6°**	**9°**	**13°**	**17°**	**20°**
Monaural listening cost	Contralateral	5.67 (3.51)	4.07 (2.85)	4.15 (3.16)	5.58 (3.84)	7.31 (4.75)	9.60 (5.09)
	Ipsilateral	7.42 (4.27)	8.91 (4.42)	10.82 (5.02)	13.59 (6.22)	16.40 (7.29)	18.23 (7.59)
Day1-Day3 Difference	Contralateral	0.59 (3.37)	0.36 (2.34)	-0.05 (3.37)	-0.13 (4.31)	-1.94 (5.10)	-1.44 (5.01)
	Ipsilateral	1.37 (3.33)	1.76 (4.14)	3.37 (3.59)	3.20 (5.11)	2.76 (5.92)	3.55 (6.04)
Day1-Day5 Difference	Contralateral	0.88 (3.60)	-0.34 (3.25)	-0.83 (3.64)	-1.45 (4.20)	-2.71 (4.98)	-2.61 (5.68)
	Ipsilateral	1.57 (4.19)	2.34 (4.55)	2.68 (4.45)	2.59 (5.17)	6.41 (6.34)	3.41 (6.02)
